# Rapid Prototyping of Personalized Articular Orthoses by Lamination of Composite Fibers upon 3D-Printed Molds

**DOI:** 10.3390/ma13040939

**Published:** 2020-02-20

**Authors:** Juan Manuel Munoz-Guijosa, Rodrigo Zapata Martínez, Adrián Martínez Cendrero, Andrés Díaz Lantada

**Affiliations:** 1 Composites and Nanocomposites Laboratory, Mechanical Engineering Department, Universidad Politécnica de Madrid; 28006 Madrid, Spain; juanmanuel.munoz.guijosa@upm.es; 2 Product Development Laboratory, Mechanical Engineering Department, Universidad Politécnica de Madrid; 28006 Madrid, Spain; r.zapata@alumnos.upm.es (R.Z.M.); adrian.mcendrero@alumnos.upm.es (A.M.C.)

**Keywords:** rapid tooling, rapid prototyping, 3D printing, 3D printed molds, fiber-reinforced polymers (FRPs), biomedical devices, articular splints, computer-aided design (CAD)

## Abstract

Advances in additive manufacturing technologies and composite materials are starting to be combined into synergic procedures that may impact the biomedical field by helping to achieve personalized and high-performance solutions for low-resource settings. In this article, we illustrate the benefits of 3D-printed rapid molds, upon which composite fibers can be laminated in a direct and resource-efficient way, for the personalized development of articular splints. The rapid mold concept presented in this work allows for a flexible lamination and curing process, even compatible with autoclaves. We demonstrate the procedure by completely developing an autoclave-cured carbon fiber–epoxy composite ankle immobilizing, supporting, or protecting splint. These medical devices may support patients in their recovery of articular injuries and for promoting a more personalized medical care employing high-performance materials, whose mechanical response is analyzed and compared to that of commercial devices. In fact, this personalization is fundamental for enhanced ergonomics, comfort during rehabilitation, and overall aesthetics. The proposed design and manufacturing strategies may support the low-cost and user-centered development of a wide set of biomedical devices and help to delocalize the supply chain for involving local populations in the development of medical technology.

## 1. Introduction

Democratization of medical technology toward universal healthcare [1] relies on innovation activities focused on minimizing the costs associated with the development of production tools, especially if personalized approaches are to be promoted. Mass-produced medical devices are obtained in many cases by means of thermoplastic injection molding within steel molds, by using precision metal stamping and forging or by employing high-speed computer numerical control machining, all of which constitute precise technologies capable of working with different biomaterials but are still too expensive for their being employed in rural or underdeveloped regions in most developing countries. Their lack of affordability makes the supply chain of the final devices more complex and prevents a delocalized manufacture, through which local populations could be involved in the decision-making steps of the healthcare industry and their economic growth could be fostered, in accordance with sustainable “blue economy” principles [2]. 

In addition, in many cases, the mass-production tools (i.e., stamps and molds) are just too expensive for application in a personalized way and can only be amortized if large series of identical devices are manufactured, hence leading to personalization in the medical industry based on ranges and percentiles, which does not provide an adequate biomechanical performance. 

Additive manufacturing, especially by means of low-cost 3D printers, which are normally based on fused-deposition modeling of thermoplastic filaments, enables designers to rapidly create complex geometries and promotes affordable device personalization in several industries, including the biomedical one, due to the need for complex shapes capable of interacting with the human body, whose geometries are also intricate. However, low-cost 3D printers still do not provide the required performance for a wide set of relevant medical devices, such as articular splints, ergonomic aids, and implants, due to the lack of mechanical properties and intrinsic anisotropy when compared to alloys or even reinforced polymers or composite materials in general. Composite materials are, in fact, starting to be considered as very attractive materials for the biomedical industry due to their excellent relationship between strength and density and between fracture energy and density, which lead to lightweight parts with high stiffness, strength and endurance. Natural-fiber-reinforced polymers and carbon-fiber-reinforced polymers are among the straightforward choices for the near future [3,4]. Nevertheless, their manufacturing processes, even if adequate for the promotion of personalized design strategies, are still too dependent on handicraft production and on the year-long expertise of qualified workers, to make them a choice toward low-cost personalized biodevices and toward local production in low-resource settings. 

Fortunately, interesting synergies between additive manufacturing and composite materials have been recently developed and may support the medical device industry to advance toward the affordable and personalized production of complex shapes, although the fused filament fabrication of composites still needs improvements for matching the performance of fiber-reinforced polymers (FRPs) obtained using traditional fabrication approaches [5]. In connection to such improvements, it is important to mention very recent advances in the 3D printing of continuous carbon fiber/epoxy composites [6] and pressure-assisted processing of continuous carbon fiber/PLA (poly(lactic acid) composites [7,8], which provide highly interesting results in terms of attainable geometrical complexity and achievable performance, even if systematic parameter testing and optimization are required. These additive processes may synergize with ongoing research in the field of innovative epoxy resins [9] and epoxy nanocomposites [10], which can benefit from special processing techniques for achieving enhanced mechanical performance for a wide set of application fields [11].

In the present study, as a complement to aforementioned advances, we describe and demonstrate the potential of 3D-printed rapid molds, upon which composite fibers can be laminated in a very straightforward and economic way, for the personalized development of articular splints or orthoses. In this case, we opt for an ankle immobilizing, supporting, or protecting splint, which is personalized to the leg of one of the participant researchers (selected as healthy volunteer) for this proof of concept. These medical devices are relevant for helping patients recover from articular injuries, and their personalization is fundamental for enhanced ergonomics and comfort during recovery and even improved aesthetics. In the following section, the materials and methods are described, before detailing and discussing our main results and discussing future challenges.

## 2. Materials and Methods 

### 2.1. Design Methods, and Open-Source and Proprietary Software

#### 2.1.1. Three-Dimensional and Personalized Ankle Digitalization

In order to reach the personalized creation of ankle supporting, immobilizing, or protecting splints, an adequate and precise approach consists of starting with a 3D digitalization of the patient’s or user’s leg as input for the CAD procedure and as an alternative to more rudimentary or manual processes based on taking some key measurements upon the user’s or patient’s ankle or foot (i.e., distance between calcaneus and phalanxes, size of fibula and tibia, among other options). 

Open-source alternatives—for non-commercial use—combining easily accessible and low-cost hardware with specialized software programs, co-exist and may support the three-dimensional reconstruction of external corporal geometries, in this case, the lower part of the leg of the final user or patient. 

Among these, it is important to mention the combined use of Microsoft Kinect for the Xbox360 (Microsoft Corporation, Redmont, WA, USA) with Skanect 3D scanning software (Occipital Inc., San Francisco, CA, USA), especially developed for working with systems such as the Kinect, Asus Xtion, or Structure Sensor, which provided the possibility of exporting to different CAD file formats, including .stl, .obj, .ply, and .vrml, directly from the images obtained with the mentioned imaging systems [12,13]. These combinations have proven to be interesting in the development of several personalized products, including medical devices [14,15]. 

Other alternatives may resort to the use of photogrammetry techniques and related software for recovering the exact positions of surface points using photographs as input. Several options exist, many of them working as apps within smartphones, depending on the size and type of object to reconstruct and on the required precision [i.e., SnapChat for face reconstructions]. 

In this study, we rely on the straightforward and versatile combination of Kinect hardware and Skanect software to reconstruct the leg of our healthy volunteer, in order to validate the proposal. These resources do not only reconstruct the 3D geometries of the leg, ankle, and foot, but also link them to additive manufacturing resources via .stl files. Once the .stl file is generated, we use Autodesk Meshmixer as support software for improving mesh quality and obtaining a softer refined surface (with an additional number of elements), which is used as input for the computer-aided design of the personalized splints and related molds, as detailed in the following subsection.

#### 2.1.2. Computer-Aided Design of Personalized Splints and Related Molds

**Figure 1:**
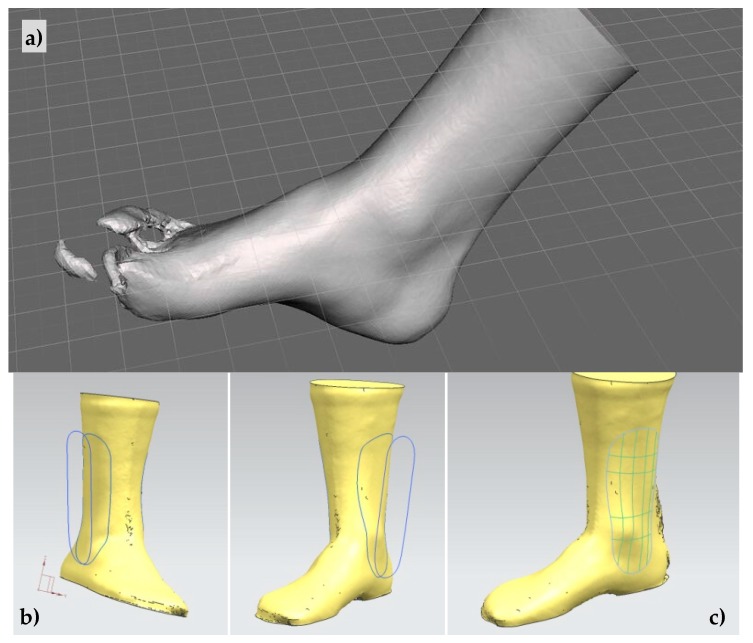
(**a**) Computer-aided design process of personalized ankle splints or orthoses. (**b**) 3D reconstruction of ankle. (**c**) Spline projections to obtain surfaces for CAD modeling.

**Figure 2:**
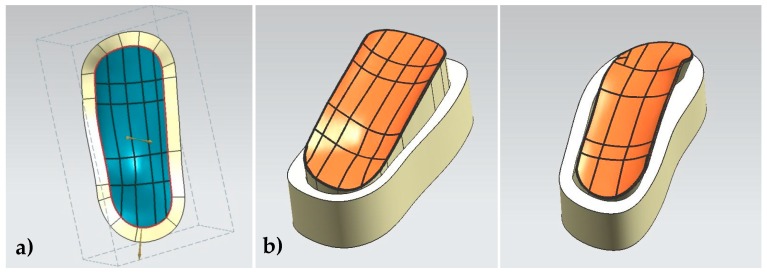
Mold design process based on the personalized ankle splint CAD models. (**a**) Partition or external surface. (**b**) Solid molds according to ankles’ surfaces.

In this study, we opt for NX-11 (Siemens PLM Software, Plano, TX, USA) as the computer-aided design software of choice due to its very special features for generating surfaces and interacting with .stl models. After importing the .stl file of the three-dimensionally reconstructed leg (Figure 1a) and adequately scaling the file to the real size of the volunteer involved in the study, the design of the personalized splint starts: 

First, a couple of planes parallel to the longitudinal cross-section of the leg are created, and two sketches with the desired lateral proportions of the two supporting or protecting splints are drawn upon them (Figure 1b). We opt for a design based on two splints, one for the internal protection of the ankle and one for the external support. Then, the sketches are projected upon the surface of the .stl reconstruction to create the profiles of the splints upon the real leg surface (Figure 1c). With the support of surface design tools of NX, the surfaces of the protecting splints are created and the “thicken” tool is used for obtaining solid and printable objects (Figure 2a). Additional “pocket” and “hole” operations are used upon these solid models to obtain the desired design (.prt file) (Figure 2b), and the conversion to an .stl file enables connection to low-cost 3D printing systems or, in general, almost all additive manufacturing technologies.

Regarding the design of alternative mold options, following the core–shell approach typical of injection molding systems, we start with the surfaces of the personalized splints by closing the holes designed for the Velcro joining straps and by extending the boundaries of the surfaces to define the partition surfaces (using “sew” and different available surface design and modification tools). We use the generated partition surfaces to cut the blocks of material toward the two required tools, which can be used for lamination. 

Final fine-tuning design operations, like the incorporation of some extrusions, for helping to laminate in the area surrounding the holes needed for the Velcro joining straps, or some small cavities for enabling the use of screwdrivers and tools for helping with the demolding process after lamination, lead to the final computer-aided designs of the personalized molds. 

The complete design processes, both for the splints and for the related molds, are presented schematically in Figure 1 and Figure 2. 

### 2.2. Manufacturing Procedures and Materials 

#### 2.2.1. Proof-of-Concept Personalized Ankle Immobilizing, Supporting or Protecting Splints

Fused deposition modeling works thanks to a heated extruder that creates superimposed layers of thermoplastic polymer by depositing lines of molten polymer filament and is inarguably the most common and affordable additive manufacturing technology, popularized worldwide under the term “3D printing”. 

In order to obtain preliminary proof-of-concept personalized ankle splints to check the ergonomics and validate the potential of the aforementioned personalized design process, a BQ Prusa Hephestos fused deposition modeling (FDM) 3D printer (BQ, Mundo Reader S.L., Las Rozas, Madrid, Spain), which constitutes an evolution of the Prusa i3 system, is used with a black PLA filament (Proto-pasta, Proto Plant, makers of Proto-pasta12001NE 60th Way, Suite B-2 Vancouver, WA 98682) as printing material.

#### 2.2.2. Rapid 3D-Printed Molds for Lamination and Autoclave Curing of Composite Fibers

Molds for lamination and curing of FRPs must fulfill several geometrical, mechanical, and thermal specifications, related to the final properties required for the cured FRP splint. First, the mold must be able to withstand the mechanical loads related to the contraction suffered by the laminate during the curing process, as well as those produced by the high curing pressure, up to 0.8 MPa, if autoclave curing is used, or up to 0.1 MPa if a vacuum-assisted infusion procedure along with an ambient curing temperature resin is used. 

In addition, the mold must maintain the required stiffness and strength at the curing temperature, which may be as high as 180 °C if autoclave curing is carried out. The mold mean surface roughness *Ra* must be small (on the order of 0.5 microns) for obtaining adequate esthetical and/or ergonomic properties at the splint visible and skin contact surfaces. These properties cannot be directly obtained with the materials usually employed in fused deposition modeling, which, on the other hand, would allow for a rapid and low-cost manufacture of the desired molds. 

Consequently, particular design considerations must be taken into account: First, we print the outer shells of the mold. We use a more precise dual-extruder BCN3D Sigma machine (BCN 3D Technologies, c/Esteve Terradas, 1, 08860, Castelldefels, Barcelona) with an acrylonitrile butadiene styrene (ABS) filament from Proto-pasta, white in this case, as printing material. If vacuum- or pressure-assisted curing is to be used, the shell containing the lamination surface must include additional, flat areas for the subsequent attachment of the supplementary materials needed, as release films, breathers, vacuum valves, and sealing tape. 

Once the outer shells of the molds are printed, a coating of epoxy resin is applied on the lamination surface in order to reach the required roughness. Subsequently, minor gaps between printed layers are closed with the help of adhesive tape, which is needed for the next process. After fine tuning of the printed mold shells, they are filled with a plaster slurry (liquid), which is hardened and dehydrated by placement in an oven at 50 °C for two hours. We use 1 and 0.5 °C/min as slopes for the heating and cooling ramps, respectively. 

This filling provides a relevant improvement in terms of mechanical endurance and of heat absorption capacity, as required for the final lamination of composite fibers, either inside the autoclave or at ambient pressure and temperature. The thermal and mechanical properties rely on the plaster core and the surface roughness properties on the epoxy coating, the ABS shell being the means for shaping the desired geometry. For this reason, the thickness of the ABS shell must be as small as possible. In the presented case, a thickness of 1 mm is achieved in the mold shells and with the 3D printer and materialemployed. 

#### 2.2.3. Lamination and Curing of FRPs toward Final Articular Splints

We have used a carbon fiber/epoxy prepreg as the lamination material, and autoclave curing for the demonstration of the process, as it is the most complex processing scheme. 

However, the presented methodology can be easily adjusted to work with less complex and more economical processes, such as a hand-lay-up of manually polyester resin-impregnated long fiberglass laminates or chopped strand mats. The prepreg laminate used is Toray T700S epoxy with a surface weight of 200 g/m^2^ and a ν_F_ of 60%. 

The lamina thickness is approximately 0.2 mm. Each lamina is manually cut by means of special fiber scissors so that the approximate final shape required for lamination is achieved and carefully placed at the mold free surface and compacted by means of a compaction roll. Scissors are again used to remove the excess material and conform the splint edges as approximate as possible to the final desired shape. Small patches are cut for achieving a good geometrical accuracy in the complex zones.

Once lamination is finished, a peelply is positioned over the outer lamina. A release film and a breather ply are positioned afterward. Finally, a vacuum bag is attached using tacky tape at the surface edges. The vacuum bag is perforated for connecting the vacuum valve to the vacuum tube. The whole set is introduced in the autoclave and conducted to a curing cycle, as required for bonding the laminated carbon fiber–epoxy sheets, with a curing temperature of 120 °C and a curing pressure of 5 bar for around 2 h. Temperature ramps of 3 °C/min are used for both the heating and cooling stages. After curing, the laminate is carefully separated from the mold and the edges are cut for its fine tuning to the desired shape. 

### 2.3. Mechanical Testing and Comparative Study

In order to validate their functionality, stiffness measurements are carried out in two replicas of epoxy–carbon fiber composite and customized ankle splints. The splints are manufactured again by the method described in Section 2.2.3, in one case using 4 laminated layers, and employing 7 laminated layers in the other, to compare in terms of weight and stiffness. A commercial ankle splint (Zetiling), whose size is selected to fit the ankle of the same individual for whom the customized splints were designed, is purchased and tested as well, again for comparative purposes with commercially available devices. 

An MTS 835 system is used for the force–displacement measurements. Crosshead speed is set to 2 mm/min. For each splint, a force-deflection measurement is carried out in a cantilever configuration, which represents a worst-case use scenario. Two additional force-deflection measurements are performed in a simply supported configuration at their central point and ankle plateau, as can be appreciated from results detailed in the following section.

## 3. Results

### 3.1. Main Results

**Figure 3:**
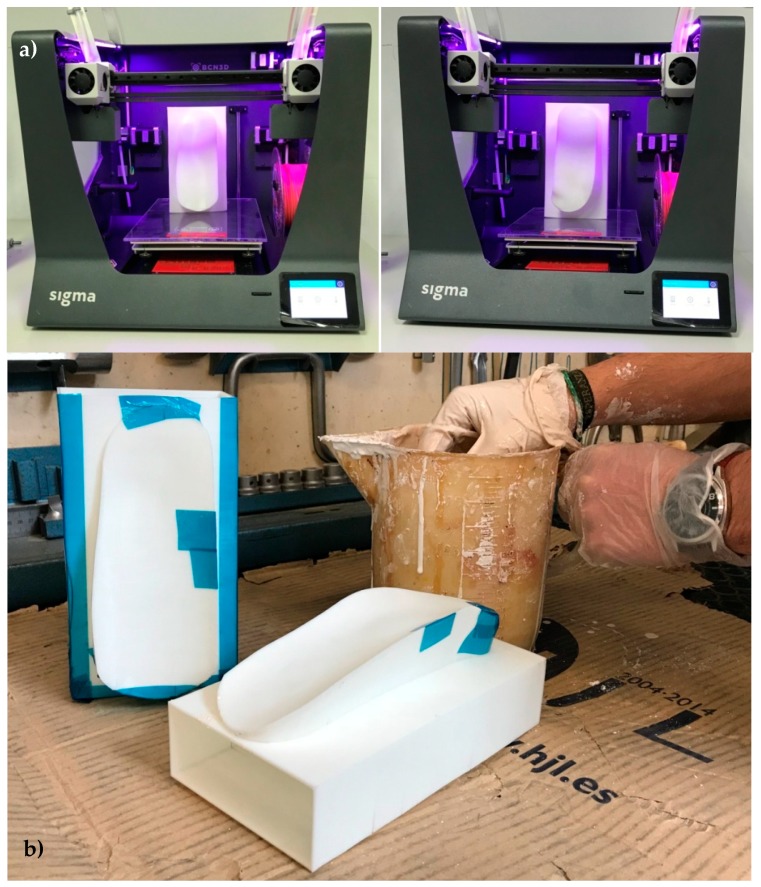
Rapid mold manufacture procedure by 3D printing and clay filling: Two molds are used for the double-sided ankle splint aimed at improved immobilization. (**a**) 3D printing. (**b**) Filling with clay.

**Figure 4:**
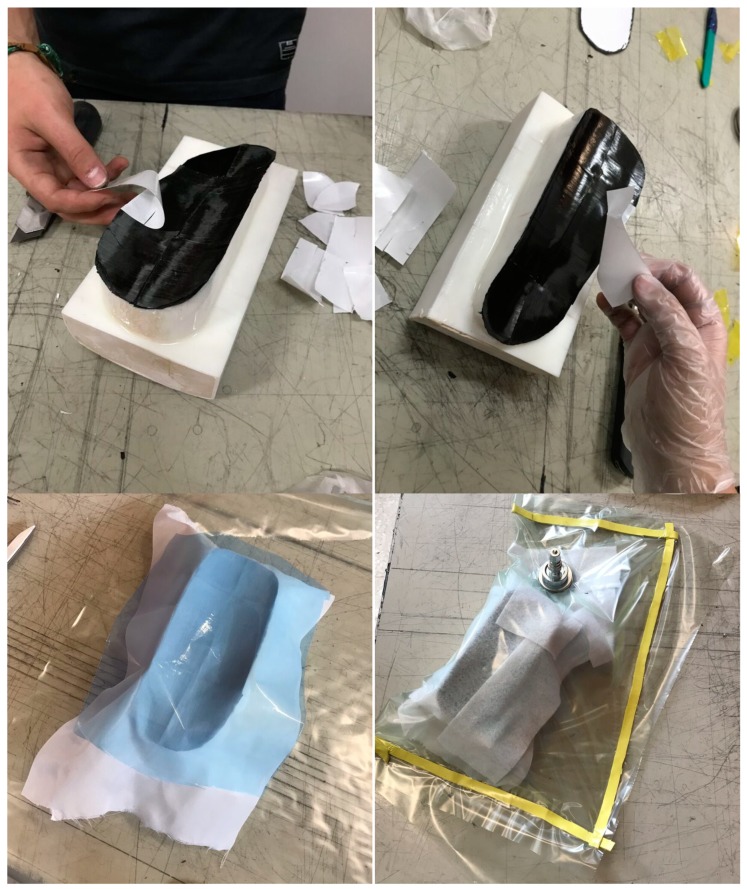
Lamination of composite fibers upon 3D-printed molds and pressurization during curing toward the final personalized orthoses.

Main results of the proposed design and manufacturing processes are presented in the following Figure 1, Figure 2, Figure 3 and Figure 4. Figure 1 presents schematically the already described process for generating a solid and personalized computer-aided design (CAD) file with the geometry of the desired ankle-supporting splints, which mainly includes the import of the three-dimensional ankle reconstruction, the creation of a planar sketch, its projection upon the ankle surface, the tessellation of a surface, and its thickening for obtaining a final printable solid. 

Figure 2 shows the creation of CAD models of different mold alternatives, which include the features of the personalized supporting splints and may serve as input for additive manufacturing technologies (a low-cost 3D printer in our case), which leads to the physical construction of rapid molds, upon which the final supporting, immobilizing, or protecting splints can be laminated. 

Figure 3 schematically shows the rapid mold manufacture procedure by 3D printing and subsequent clay filling. Two molds for the double-sided ankle splint aimed at improved immobilization are presented. The outer shells of the molds obtained within the FDM 3D printer are highlighted (Figure 3a). Some Scotch-tape patches can be seen in the mold shells for covering minor holes and manufacturing defects of the 3D printing process, hence preventing leakage during the clay filling process (Figure 3b). 

**Figure 5:**
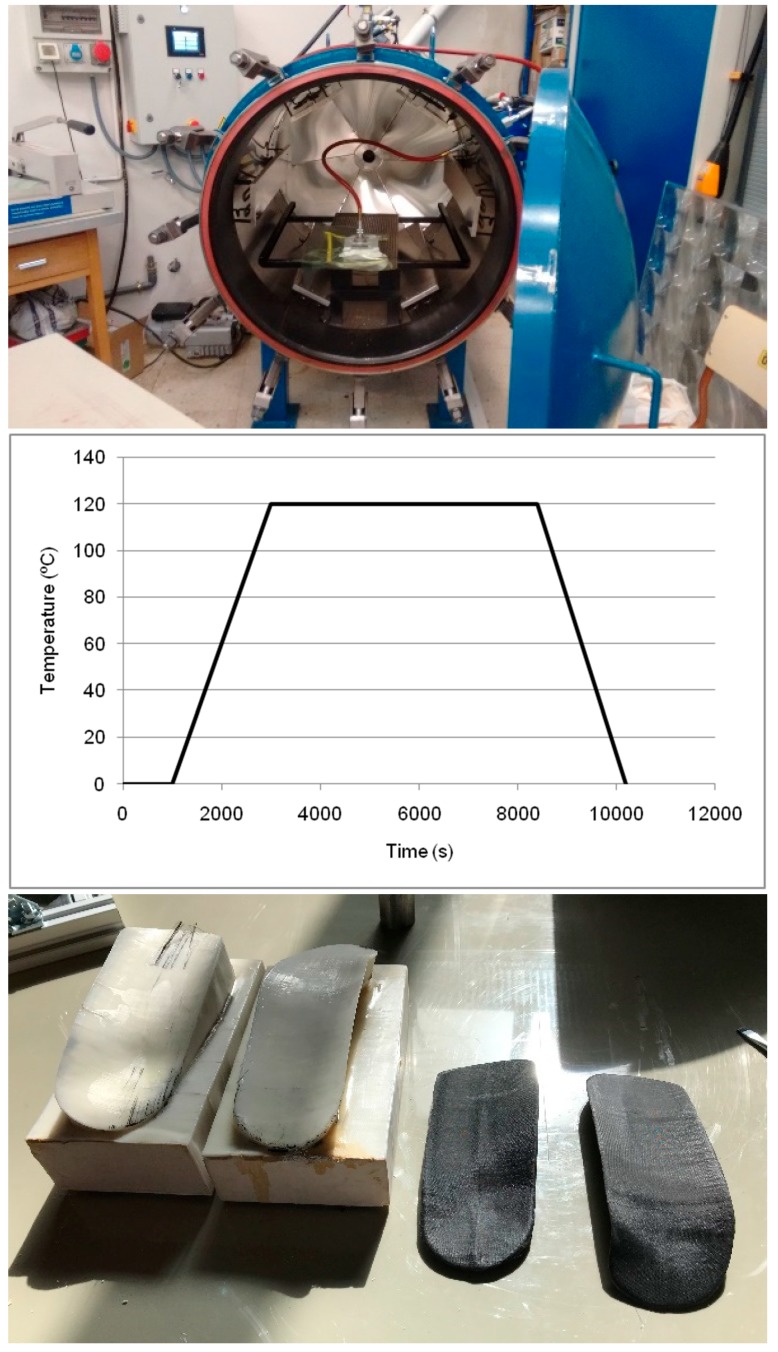
Autoclave with applied temperature cycle and finally obtained composite ankle splint structures and reusable molds.

**Figure 6:**
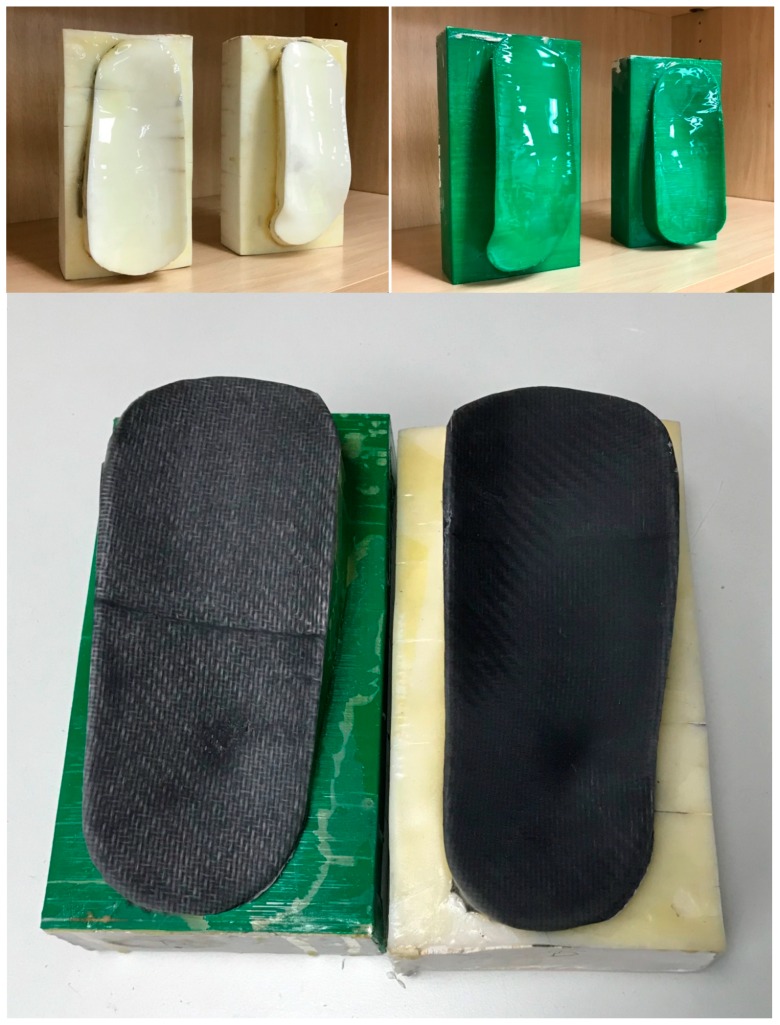
Final 3D-printed molds with the obtained personalized ankle splints in carbon fiber.

**Figure 7:**
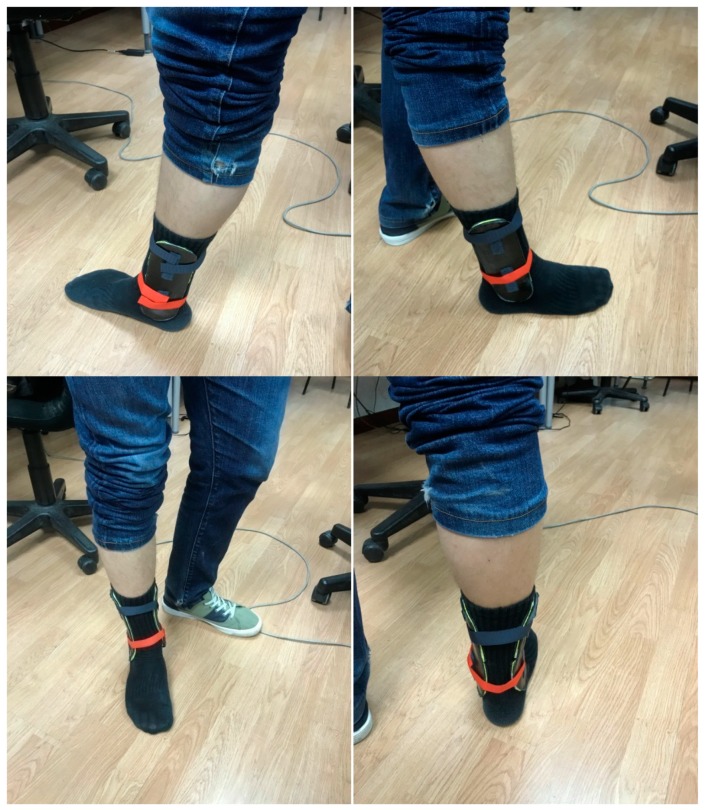
Evaluation of ergonomic performance upon healthy volunteer, after adding inner glued soft protective pads and fixation strips to the carbon fiber splint structures.

The final mold is polished and coated with epoxy resin, upon which the composite fibers are laminated and subject to vacuum, as schematically presented in Figure 4, and then autoclaved, as shown in Figure 5. The final ankle splint structure in the composite material is presented in Figure 6, together with the 3D-printed mold after the production process, and an ergonomic evaluation upon a healthy volunteer is provided in Figure 7. It is important to highlight that the final articular orthoses or splints, despite requiring a final polishing and minor adjustments for enhanced comfort, stand out for their structural integrity, stiffness, low thickness, and overall lightweight structure, when compared to the 3D-printed proof-of-concept models.

### 3.2. Discussion

The manufacture of molds and prototypes has helped to obtain data regarding manufacturing time, involved costs, mold roughness, and molding cycles with the developed manufacturing tools (printed molds) and has been compared to common state-of-the-art processes. The comparative is included in Table 1. In short, the proposed process stands out for the cost reduction when compared to industrial processes relying on metallic molds, especially for the purpose of personalized biodevices, which do not require replication beyond 10 copies. The time required for creating the tools is on the same order of magnitude as in the case of metallic molds, while the cost is at least one order of magnitude lower. 

In addition, the possibility of obtaining these manufacturing tools for the personalized production of composite parts, by using conventional 3D printers and low-cost materials, proves interesting for working in remote regions and low-resource settings, in which limited access to technology and challenges linked to the supply chain of materials and resources may prevent equitable access to healthcare technologies and medical devices. With this procedure, personalization and point-of-care manufacture, even in campaign hospitals with just a small “fab-lab,” is possible. 

In connection to this, sharing processes and project results through open-access and collaborative research e-infrastructures may help with the progressive democratization of healthcare technology. Accordingly, the results of the study are shared through the UBORA e-infrastructure (https://platform.ubora-biomedical.org/), to allow technology developers and healthcare professionals to benefit from these advances or to use these developments as case studies for training purposes in the field of biomedical engineering or in connection with educational activities for healthcare professionals [16].

It is important to consider that, even if master models, rapid prototyped molds, and sacrificial tools have already been proposed for manufacturing composites, they normally rely on fused deposition modeling of high-performance polymers, such as Ultem [18], for which special 3D printers are needed. Ultem printers can be found in typical price ranges of €5000–€20,000, while more conventional low-cost 3D printers, as the one used in this study, can be found in the €500–€2000 range, an order of magnitude lower than adequate for Ultem. The rapid tooling process presented here is innovative as it resorts to conventional fused deposition modeling of common thermoplastics while improving the mechanical properties of the generated tools by filling them with clay, which proves adequate for lamination and autoclave curing of the epoxy–carbon fiber sheets. Hence, the process has potential for spreading in low-resource settings, where common FDM printers are found.

**Figure 8:**
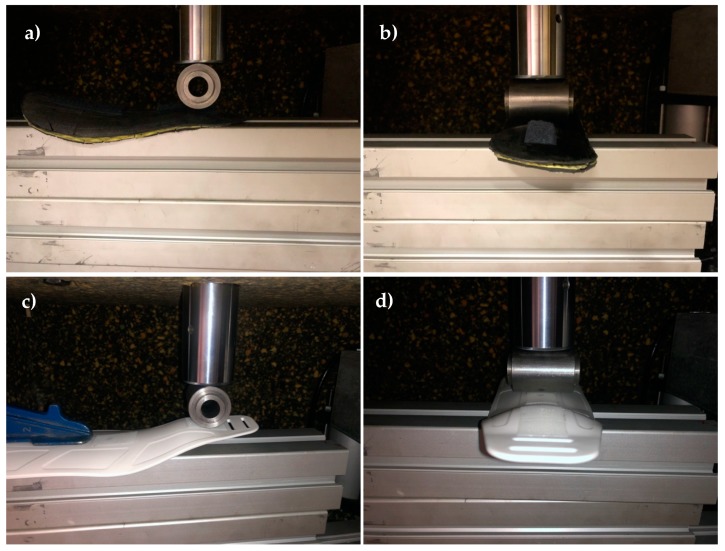
Stiffness measurement in (**a**,**c**) cantilever and (**b**,**d**) simply supported configurations.

**Figure 9:**
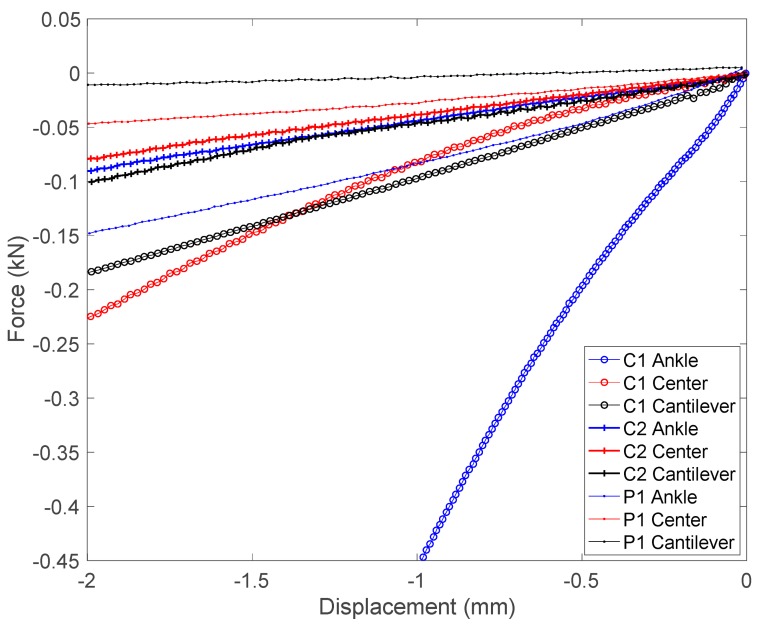
Splint force–displacement graph, showing testing results, both for the carbon fiber–epoxy composite customized ankle splints (C1 and C2) and for a commercial polymeric splint (P1).

In terms of mechanical performance, Table 2 summarizes the main characteristics of each of the splints tested, the two customized ones manufactured with the carbon fiber–epoxy composite, and the commercial one, as advanced in Section 2.3. An MTS 835 system is used for the measurements with the crosshead speed set at 2 mm/min, as already mentioned. For each of the splints, a force-deflection measurement is carried out in the cantilever configuration (shown in Figure 8a,c), which represents a critical use scenario. Two additional force-deflection measurements are performed in a simply supported configuration (shown in Figure 8b,d) at their central point of the splints and at the ankle plateau. Figure 9 shows the measurement results. As expected, stiffness is substantially influenced by the splint thickness, which allows for a fine tuning of the splint characteristics. As observed in the summarizing graph, the C1 splint, with seven layers and a thickness of 2.1 mm, has a clearly higher stiffness than the commercial one (P1) and a similar weight. 

In the case of the C2 splint, comprising four layers and with a thickness of just 1.2 mm, the mechanical behavior is very similar to that observed in the commercial, with less than half of its weight. Furthermore, mechanical testing helps to put forward that the variation in the number of laminated sheets can help to fine-tune the mechanical performance of these composite splints, depending on the final application (i.e., from articular immobilization, to impact protection, among others). Besides, the rapid tools used prove to be adequate for replicating several splints for the same patient, although reshaping is more complex and may still need gel-filled or inflated adapters, as conventional splints do.

## 4. Current Challenges and Future Proposals

Regarding current challenges, we would like to mention that the proposed rapid prototyping processes can be combined with synergistic technologies and procedures, such as casting or injection of soft materials within rapid molds, in which the composite parts are placed as inserts, to obtain final devices with functional gradients of stiffness for enhanced comfort and ergonomics. A systematic analysis of potential risks involved in using composite parts as protecting or immobilizing elements in medical appliances, together with associated redesign activities oriented to minimizing detected risks, would be needed, as part of pre-production activities leading to the eventual commercialization of these types of solutions. 

Evaluation in accordance with ISO standards “10993 on the Biological Evaluation of Medical Devices” and “14971 on the Application of Risk Management to Medical Devices,” to demonstrate compliance of these articular splints with EU Medical Device Regulation 745/2017, is still needed, as this study is focused on the development and validation of an innovative rapid prototyping process by means of non-commercial prototypes. As for the future, working toward the standardization of these processes and for their progressive incorporation into the medical device industry, as a reference option for the creation of personalized composite splints, and validating them with patients, not only in remote locations and low-resource settings but also as part of common procedures in hospitals and primary care centers, are the main proposed continuation lines. 

Synergies between standard industrial processes for the production of composite parts and rapid prototyping tools, to steadily adapt high-performance production tools to the demands of industries and appliances requiring personalization (i.e., by using 3D-printing inserts within multipurpose industrial frames and tools), should also be further explored.

## 5. Conclusions

This study illustrates recently developed manufacturing processes, in which the advantages of low-cost additive manufacturing and composite materials are combined in a synergic way, by providing a complete development case of study linked to the personalized development of articular splints or orthoses for improved injury recovery. By means of example, an ankle immobilizing, supporting, or protecting splint is developed in a personalized way, adjusting it to the ankle of one of the participant researchers of the study. The procedure stands out for its affordability and personalized focus, which opens new horizons in the biomedical industry toward the delocalization of supply chains and the progressive involvement of local populations in low-resource settings, both during the decision-making steps and during the implementation and operation phases, which may not only bring just personalized and cheap high-performance solutions, but also improve the local economies. The information and files of the complete case of study are accessible through the UBORA e-infrastructure, a sort of “Wikipedia” of medical devices (please access: following website https://platform.ubora-biomedical.org and check project called: “Universal 4D printed personalized splints for articular pathologies”), in connection with the open-source approaches to medical device development and to biomedical engineering in general that our team is currently pursuing within the UBORA project.

## Figures and Tables

**Table 1:** Comparative study of time and cost devoted to creating the personalized splints: Actual data from present study and estimation of conventional handicraft procedures * [17].

Mold Type	Manufacturing Time (h)	Cost (€)	Roughness (μm)	Molding Cycles
Machined aluminum	~20–30	~1500	0.4	>30,000 *
3D-printed ABS coated with epoxy	45.5	47.36	0.5	>5
Polished ABS after 3D printing	41	40.58	3.6	1–2

**Table 2:** Representative data of different splints employed for mechanical validation.

Splint	Material	Joint	Number of Layers	Thickness (mm)	Weight (g)
Customized 1	T700 epoxy prepreg, 300 g/m^2^	Ankle	7	2.1	53.5
Customized 2	T700 epoxy prepreg, 300 g/m^2^	Ankle	4	1.2	24.0
Commercial	Thermoplastic polymer	Ankle	–	2.5	54.5
